# Casticin attenuates liver fibrosis and hepatic stellate cell activation by blocking TGF-β/Smad signaling pathway

**DOI:** 10.18632/oncotarget.17453

**Published:** 2017-04-27

**Authors:** Ling Zhou, Xiaoying Dong, Linlin Wang, Lanlan Shan, Ting Li, Wanfu Xu, Yan Ding, Mingqiang Lai, Xiaojun Lin, Meng Dai, Xiaochun Bai, Chunhong Jia, Hang Zheng

**Affiliations:** ^1^ Department of Oncology, Nanfang Hospital, Southern Medical University, Guangzhou, Guangdong, China; ^2^ Department of Health Management, Nanfang Hospital, Southern Medical University, Guangzhou, Guangdong, China; ^3^ Department of Hepatobiliary Surgery, Zhujiang Hospital, Southern Medical University, Guangzhou, Guangdong, China; ^4^ Department of Gastroenterology, Guangzhou Women and Children's Medical Center, Guangzhou Medical University, Guangzhou, Guangdong, China; ^5^ Department of Cell Biology, School of Basic Medical Sciences, Southern Medical University, Guangzhou, Guangdong, China

**Keywords:** casticin, CCl_4_, liver fibrosis, hepatic stellate cell, TGF-β/Smad

## Abstract

Although many advances have been made in understanding the pathogenesis of liver fibrosis, few options are available for treatment. Casticin, one of the major flavonoids in *Fructus Viticis* extracts, has shown hepatoprotective potential, but its effects on liver fibrosis are not clear. In this study, we investigated the antifibrotic activity of casticin and its underlying mechanism *in vivo* and *in vitro*. Male mice were injected intraperitoneally with carbon tetrachloride (CCl_4_) or underwent bile duct ligation (BDL) to induce liver fibrosis, followed by treatment with casticin or vehicle. In addition, transforming growth factor-β1(TGF-β1)-activated LX-2 cells were used. *In vivo* experiments showed that treatment with casticin alone had no toxic effect while significantly attenuating CCl_4_-or BDL-induced liver fibrosis, as indicated by reductions in the density of fibrosis, hydroxyproline content, expression of α-SMA and collagen α1(I) mRNA. Moreover, casticin inhibited LX2 proliferation, induced apoptosis in a time- and dose-dependent manner *in vitro*. The underlying molecular mechanisms for the effect of casticin involved inhibition of hepatic stellate cell (HSC) activation and reduced the expression of matrix metalloproteinase (MMP)-2, MMP-9, tissue inhibitor of metalloproteinases (TIMP)-1 and TIMP-2 resulting from blocking TGF-β1/Smad signaling, as well as increased the apoptosis of HSCs. The results suggest that casticin has potential benefits in the attenuation and treatment of liver fibrosis.

## INTRODUCTION

Liver fibrosis is characterized by the excessive deposition of extracellular matrix (ECM) components, mainly fibrillar collagen, which occurs in most types of chronic liver diseases [1]. This pathological status is the result of a dynamic process usually preceded by liver injuries such as chronic inflammation, viral hepatitis, alcohol abuse, metabolic liver diseases and others [2]. If the injuries persist, fibrillar collagen accumulates, resulting in fibrosis and ultimately impairment of liver function. Advanced liver fibrosis results in cirrhosis, hepatocellular carcinoma, liver failure and portal hypertension[3, 4]. Although great progress has been made in elucidating the mechanisms underlying liver fibrosis, few options are available for its treatment [5–9].

Activated hepatic stellate cells (HSCs) are the major contributor to the production of fibrillar collagen in the injured liver [10]. In the normal liver, HSCs are quiescent, residing in the space of Disse, and function as the main storage site of vitamin A. Following chronic injury, HSCs undergo a dramatic phenotypical transformation into α-smooth muscle actin (α-SMA)-positive myofibroblast-like cells and increase their expression of fibrillar collagen and matrix metalloproteinases (MMPs) such as MMP2 and MMP9, as well as tissue inhibitors of metalloproteinases (TIMPs). Consequently, fibrillar collagen accumulates, especially collagen I and collagen III. Increasing evidence indicates that transforming growth factor beta 1 (TGF-β1) is a key mediator in this pathogenesis by activating its downstream Smad signaling pathway [11–13]. According to differences in structure and function, Smads are classified into three groups. Smad2/3 are named receptor-activated Smads (R-Smads), and Smad4 is categorized as a common Smad (Co-Smad). Smads6, 7, and 8 are inhibitory Smads (I-Smad). When TGF-β1 binds to its receptor, Smad2/3 is phosphorylated and binds with Smad4, followed by translocation into the nucleus where these complexes activate transcription of profibrotic genes [14]. Therefore, inhibiting the accumulation of activated HSCs by modulating either their activation and/or proliferation, or by promoting their apoptosis through the TGF-β/Smad signaling pathway, is a potential target for therapy [5, 15].

Many natural compounds have been shown to have hepatoprotective effects [16–18]. *Vitex rotundifolia L*. is a traditional herbal medicine in oriental countries. Its ripe fruits are called *Fructus Viticis* and have been used as folk medicine for headaches, colds, migraine and eye pain. Casticin(3′, 5-dihydroxy-3, 4′, 6, 7-tetramethoxyflavone) is one of the major flavonoids in *Fructus Viticis* extracts [19]. It has been shown to have several biological activities, but most studies have focused on its anti-tumor effects in different types of cancer [20–24]. More recently, an anti-inflammatory effect by casticin has been reported *in vitro* and *in vivo* [25]. Casticin has been shown to ameliorate cigarette smoke-induced acute lung inflammation and reduce croton oil-induced ear dermatitis and edema in mice [26]. However, its effect on liver fibrosis has not yet been examined.

Here, to elucidate the potential effect of casticin on liver fibrosis *in vivo*, casticin was administered to mice that had received carbon tetrachloride (CCl_4_) injections or undergone bile duct ligation (BDL) to induce liver fibrosis. As we hypothesized, casticin prevented the activation of HSCs and reduced liver fibrosis in these two models. In order to determine the probable mechanisms, we also investigated the effects of casticin on TGF-β1-mediated HSC activation in LX2 cells from an immortalized human HSC cell line. We confirmed that casticin attenuated liver fibrosis probably by inhibiting the HSCs activation through TGF-β/Smad signaling pathway and inducing the apoptosis of HSCs. Our results suggest the potential of casticin as a new therapeutic drug for the treatment of hepatic fibrosis.

## RESULTS

### Casticin prevents hepatic injury induced by CCl_4_ or BDL *in vivo*

**Figure 1:**
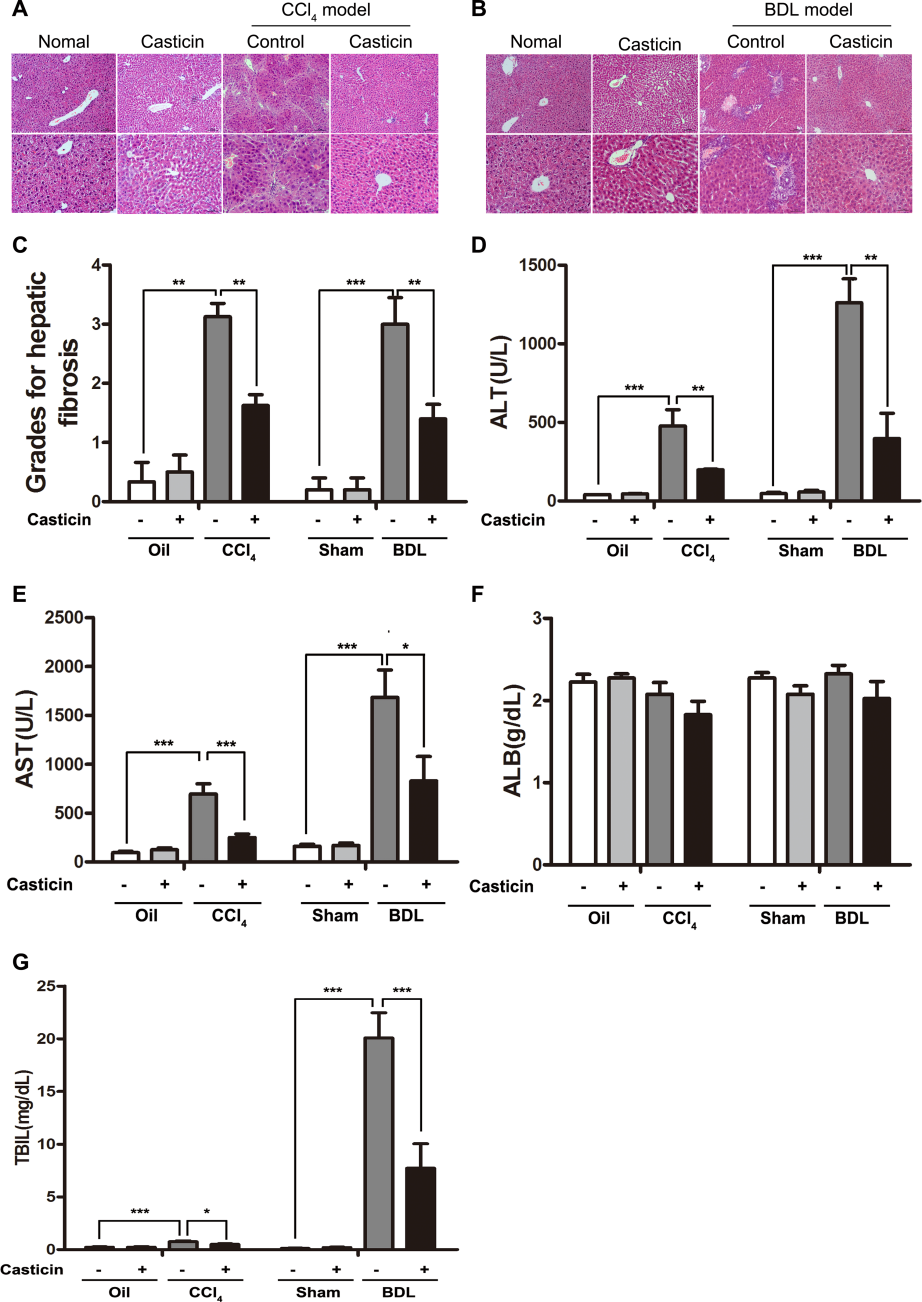
Hepatoprotective effect of casticin in CCl_4_-and BDL-induced hepatic injury Mice were given repeated injections of CCl_4_ for 6 weeks, and then given oral administration of casticin for 2 weeks. Representative photomicrographs of liver histology from normal healthy mice, casticin alone, CCl_4_ + vehicle alone and CCl_4_ + casticin (**A**) are shown. Other mice underwent BDL or sham operation for 4 days, and then were given oral administration of casticin or vehicle for 2 weeks. Representative photomicrographs of liver histology from sham, sham + casticin, BDL + vehicle alone, and BDL + casticin are shown (**B**). Grading of hepatic fibrosis was evaluated on a scale of 1–4 as follows: 1, scattered; 2, mild; 3, moderate and 4, marked. The histological changes were assessed in 200× magnification fields of slides stained with H&E (**C**). Serum aminotransferases, albumin and total bilirubin levels are plotted(**D**–**G**). The values are expressed as means ± SEM of eight determinations per group. **p < 0.01, ***p < 0.001.

The *in vivo* effect of casticin on progression of fibrosis was assessed in two experimental mice models induced by CCl_4_ or bile duct ligation (BDL). In the first model, mice were given repeated injections of CCl_4_ for 6 weeks, and subsequently casticin (20 mg/kg) was administered by gastric gavage everyday for 2 weeks after the final CCl_4_ treatment. In the second model, mice underwent either sham operation or BDL. After BDL for 4 days, mice were administered intragastrically with casticin at 20 mg/kg/day(casticin dissolved in 0.25% Tween-80) or 0.25% Tween-80 for 2 weeks. Liver specimens were obtained 24 h after the last administration of casticin, and morphological changes of liver injury and fibrosis were visualized in sections stained by H&E. As expected, thick fibrotic septa and pseudolobular formation were more extensive in mice exposed to CCl_4_ or undergone BDL compared to controls (Figure 1A–1B). Similarly, the grades of fibrosis in the CCl_4_ and BDL fibrotic models were severe than controls (Figure 1C). Moreover, serum ALT, AST, albumin and total bilirubin were elevated in the CCl_4_ and BDL groups compared to controls (Figure 1D–1G). In contrast, treatment with casticin led to attenuation of both histological and functional injury. Mice treated with casticin alone displayed normal histology and serological values similar to the control mice. It indicated that treatment with casticin alone for two weeks had no toxic effect on the liver. These observations clearly demonstrate that casticin exerted a hepatoprotective effect.

### Casticin attenuates liver fibrosis induced by CCl_4_ or BDL *in vivo*

**Figure 2:**
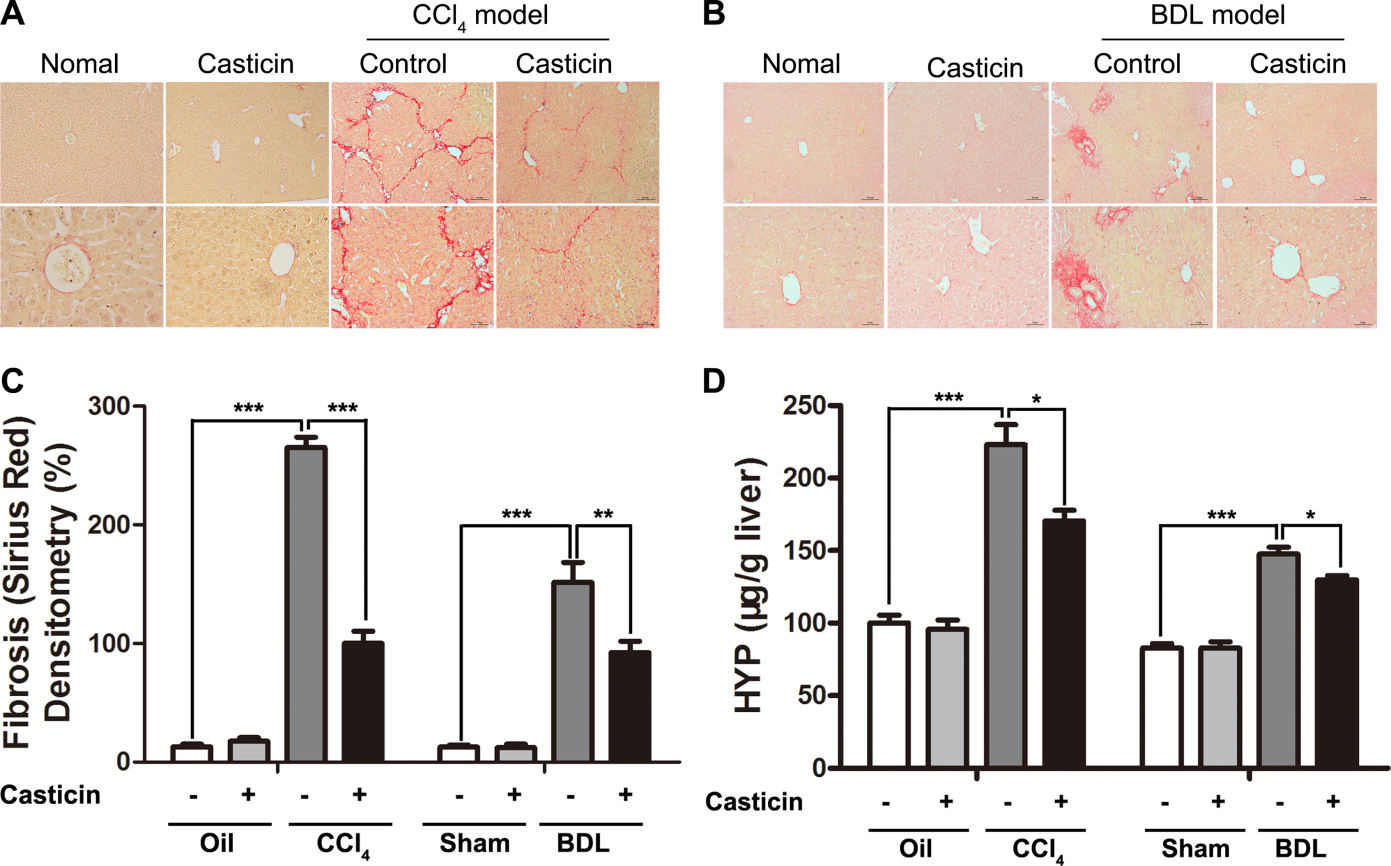
Effect of casticin on alleviation of CCl_4_-and BDL-induced hepatic fibrosis (**A**–**B**) Histology with sirius red staining of liver sections (original magnification 100× and 400×). (**C**) Relative densitometry of fibrosis in mice. The density of fibrosis was determined as intensity of sirius red staining divided by the area of the captured field. A total of 24 fields were captured from livers in each group of mice. (**D**) Liver hydroxyproline content was determined to estimate collagen content, expressed as μg hydroxyproline per g liver tissue (wet weight). The values are expressed as means ± SEM (*n* = 8). **p < 0.01, ***p < 0.001.

Subsequently, the effect of casticin on alleviation of hepatic fibrosis was observed in liver sections stained by sirius red (Figure 2A–2B). Morphometric analysis of sirius red staining demonstrated that compared with control mice, CCl_4_ or BDL treatment caused a remarkable collagen accumulation in the liver, shown in Figure 2C. In contrast, treatment with casticin markedly decreased hepatic collagen matrix accumulation. Furthermore, the administration of casticin facilitated a decrease in hepatic hydroxyproline contents (Figure 2D). Collectively, these findings indicate that casticin attenuated hepatic fibrosis induced by CCl_4_ or BDL *in vivo*.

**Figure 3:**
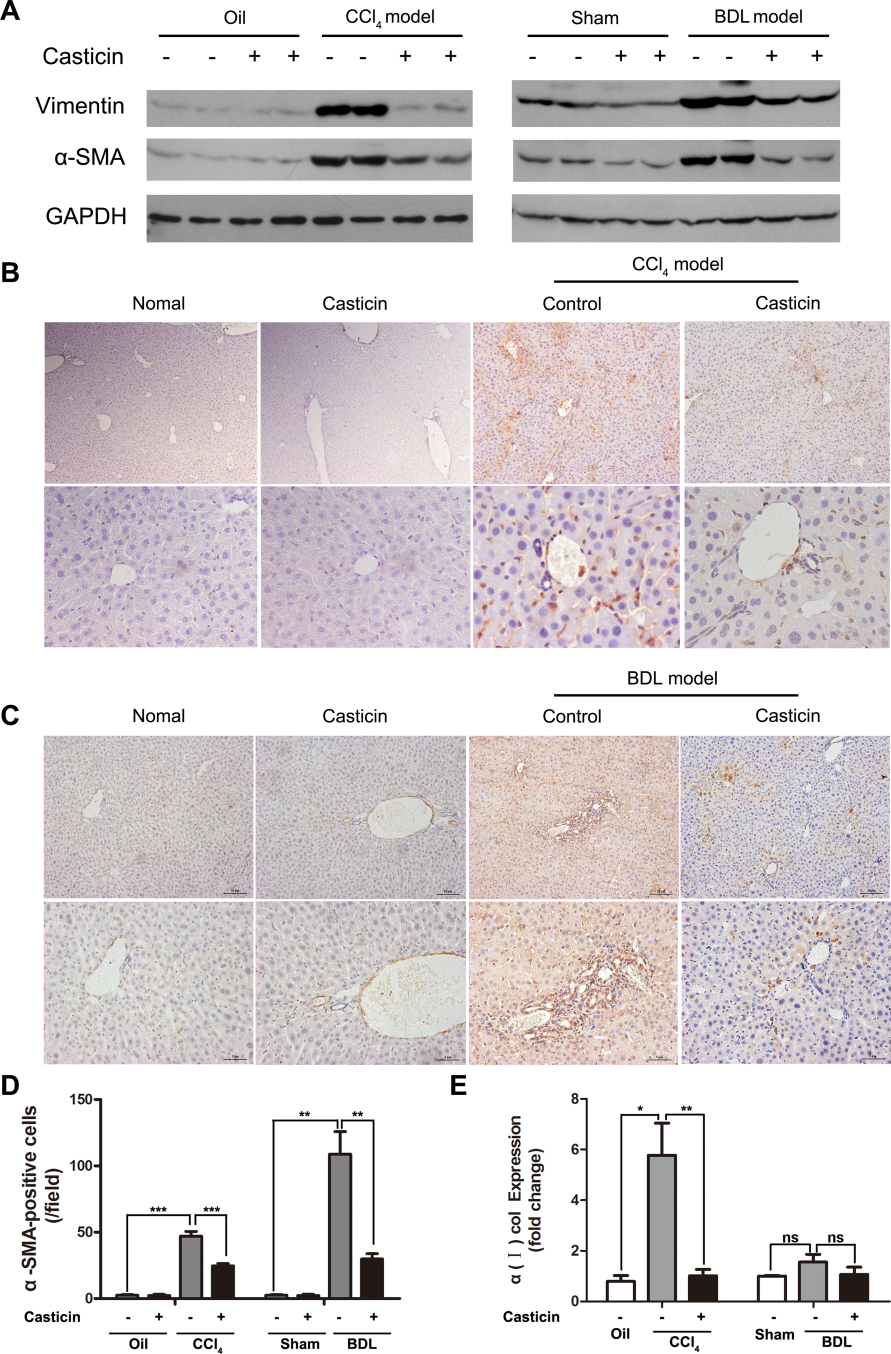
Effect of casticin on proliferation and activation of HSCs (**A**) Following the treatment previously described, the mice were euthanatized and their liver tissue was lysed and subjected to western blot analysis to assess α-SMA and vimentin. (**B**–**C**) Immunohistochemical staining of α-SMA. (**D**) Numbers of α-SMA positive cells were counted in 10 random low-power fields. Average numbers of positive cells in the fields are plotted (*n* = 8, ***p < 0.001). (**E**) Hepatic mRNA levels for collagen α1(I) were measured by real-time RT-PCR. The relative amounts of mRNA were normalized against GAPDH in the same samples (*n* = 8, **p < 0.01).

Hepatic stellate cells are a major cell type responsible for liver fibrosis following their activation into fibrogenic myofibroblast-like cells, and α-SMA is an excellent marker for detection [27]. As shown in Figure 3A, western blot analyses for α-SMA and vimentin were performed. α-SMA and vimentin protein expression were higher in the CCl_4_ and BDL groups compared with control groups, while the casticin treatment can reduce the expression sharply. In addition, immunohistochemical staining for α-SMA showed that α-SMA-positive cells were rarely detected in the control groups, but were clearly evident in the CCl_4_ and BDL groups. In sharp contrast, however, α-SMA-positive cells were markedly decreased in liver sections of CCl_4_ and BDL- injured mice treated with casticin (Figure 3B–3C). In fact, the number of α-SMA-positive cells per field was nearly 2-fold lower in the CCl_4_ + casticin group compared to the CCl_4_ group and nearly 4-fold lower in the BDL+ casticin group compared to the BDL group (Figure 3D). Furthermore, hepatic expression levels of collagen α1(I) mRNA were significantly decreased following casticin treatment (Figure 3E). Collectively, these findings suggest that casticin likely attenuated hepatic fibrosis induced by CCl_4_ or BDL *in vivo* probably by inhibiting HSC proliferation and activation.

### Casticin inhibits proliferation and induces apoptosis in LX2 cells

**Figure 4:**
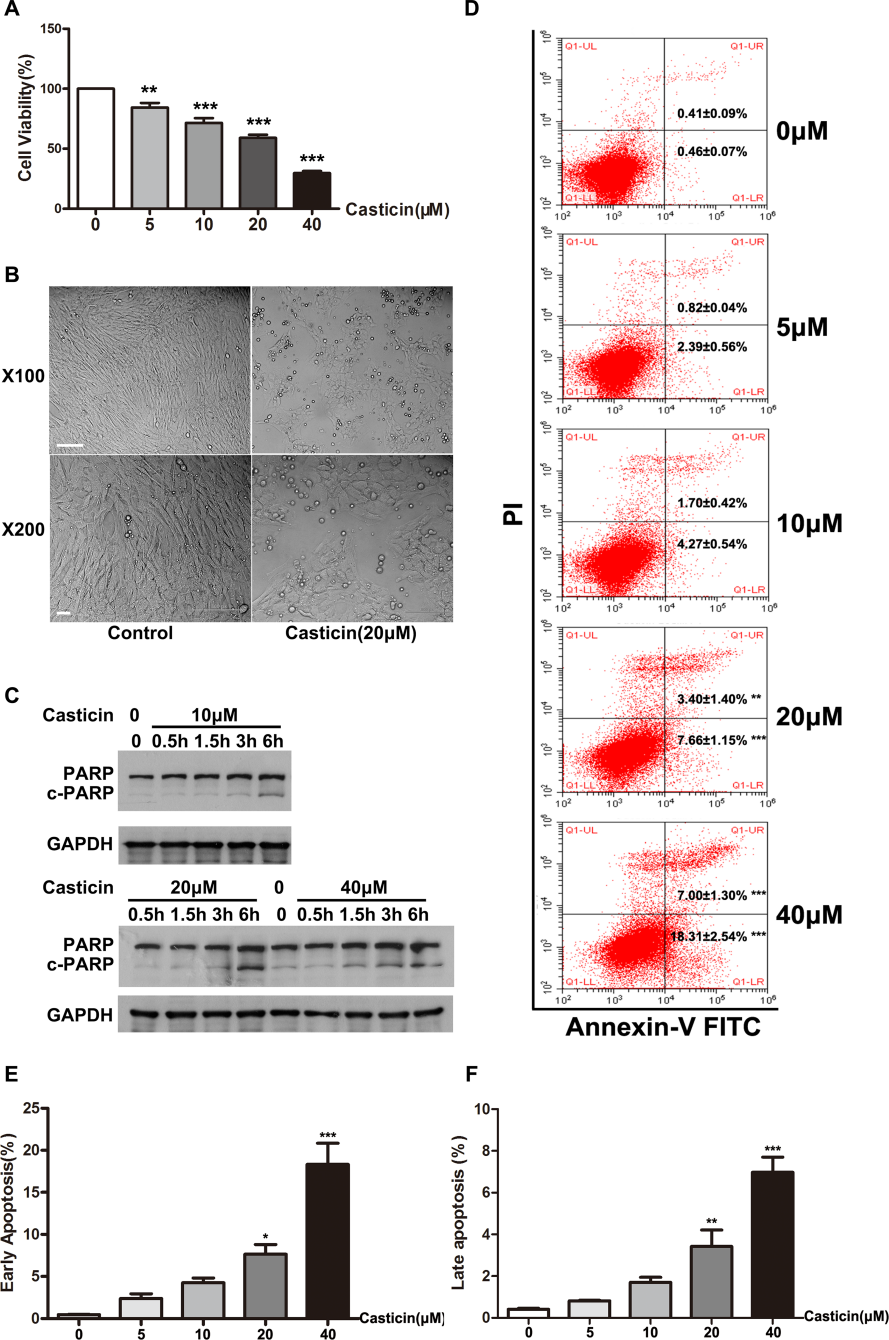
Effect of casticin on cell proliferation and apoptosis of LX2 cells (**A**) Cell proliferation was determined by CCK-8 assay. LX2 cells plated in 96-well plates were treated with casticin (5 μM, 10 μM, 20 μM or 40 μM) in growth medium containing serum for 48 h. (**B**) LX2 cells were cultured for 24 h, then incubated with casticin (20 μM) for up to 12 h. Representative phase-contrast photomicrographs of LX2 cells after incubation with equivoluminal DMSO or 20 μM casticin for 12 h are shown (original magnification 100× and 400×). (**C**) LX2 cells were cultured for 24 h, then treated with casticin (10 μM, 20 μM or 40 μM) for 0.5 h, 1.5 h, 3 h or 6 h. Cell lysates were subjected to western blot analysis to assess the cleavage of PARP. (**D**–**F**) LX2 cells were cultured for 24 h, then incubated with casticin (20 μM) for up to 12 h. Then casticin-induced cell apoptosis as measured by Annexin V–FITC and PI staining.

The LX-2 human hepatic stellate cell line has been widely characterized and maintains key features of hepatic stellate cytokine signaling, retinoid metabolism and fibrogenesis, making it a very suitable model of human hepatic fibrosis. To explore the underlying mechanisms for our *in vivo* observations, we carried out *in vitro* studies using LX-2 cells. We first confirmed that casticin inhibited proliferation of LX2 cells in a concentration-dependent manner (Figure 4A). Subsequently, the influence of casticin on LX2 cell apoptosis was assessed by morphological changes, AV-PI staining and flow cytometric assay. In the presence of 20 μM casticin, adherent LX2 cells began shrinking after 3 h; the majority of cells were detached from the dishes in 12 h (Figure 4B). And the AV-PI staining and flow cytometric assay results indicated that casticin induced cell apoptosis in a dose-dependent fashion (Figure 4D–4F). It was well known that cleavage of PARP facilitated cellular disassembly and served as a marker of cells undergoing apoptosis [28, 29]. Further, western blot analyses for cleaved PARP were performed (Figure 4C). Cleaved PARP was barely detectable in untreated LX2 cells, while specific bands corresponding to full-length PARP were clearly detected. In contrast, cleaved PARP was obviously detected in LX2 cells treated with 10 μM casticin for 1.5–6 h. Similar results were obtained in LX2 cells treated with 20 μM and 40 μM casticin. These findings strongly suggest that casticin inhibited LX-2 cell proliferation while promoting apoptosis in a time- and dose-dependent manner. Next, we explored the effect of casticin on L02 cells by cell proliferation assay and apoptosis analysis. We found that small dose(0–20 μM) casticin had no toxic effect on L02 cells. However, 40 μM casticin would suppresses L02 cells proliferation and induces apoptosis (Supplementary Figure 1B–1E).

### Casticin inhibits HSC activation and collagen matrix expression by blocking TGF-β/Smad signaling in LX2 cells

**Figure 5:**
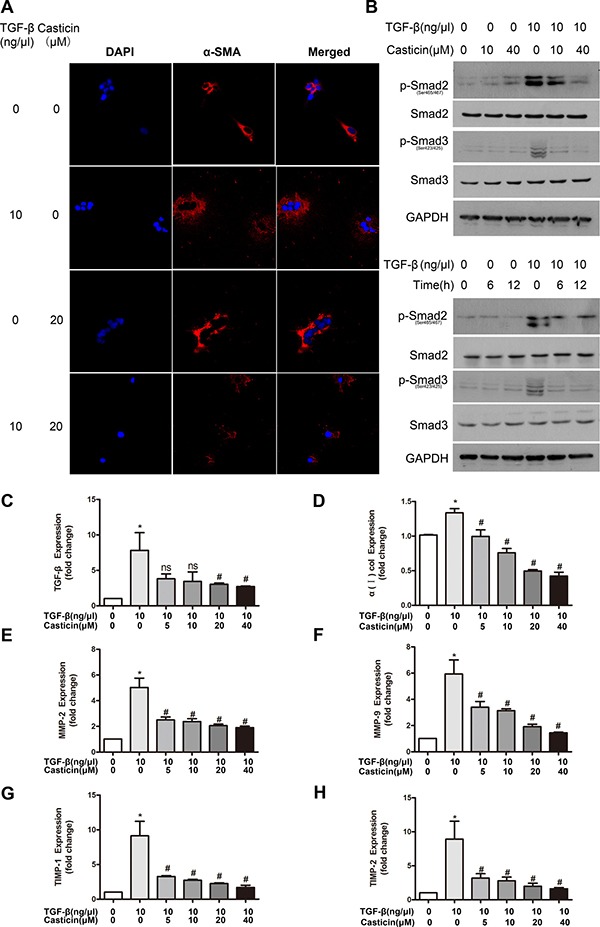
Effects and probable mechanisms of casticin on HSC activation *in vitro* (**A**) Immunofluorescent detection of LX2 cells. Immunostaining was performed to detect expression of α-SMA (red, 600×) with nuclear counterstaining by DAPI (blue). (**B**) The phosphorylation level of Smad2/3 was analyzed by western blot. LX2 cells were plated in 24-well plates, incubated in serum-free medium for 12 h, followed by treatment with casticin in various concentrations (10–40 μM) for different durations (6 h/12 h) in the presence or absence of TGF-β1 (10 ng/μl) for 1 h. Cell lysates were subjected to western blot analysis. (**C**–**H**) TGF-β, collagen α1(I), MMP-2, MMP-9, TIMP1 and TIMP-2 mRNAs were measured by real-time RT-PCR (*n* = 5). *p < 0.05 vs. control, ^#^*p* < 0.05 vs. TGF-β alone.

Since casticin inhibited cell proliferation and apoptosis, we next examined whether casticin could suppress HSC activation. It has been demonstrated that TGF-β1 is a classic activator of HSCs and a key mediator during development of liver fibrosis. Serum-starved LX-2 cells were treated with casticin for 12 h followed by TGF-β1 stimulation for 1 h. As expected, TGF-β1 increased LX-2 activation in control cells, as indicated by enhanced α-SMA expression, but this activation was suppressed in the presence of casticin. Meanwhile, the expression of α-SMA protein was assessed by immunofluorescence, which showed that the α-SMA expression increase under the TGF-β1 treatment, but this increase was attenuated by treatment with casticin (Figure 5A).

Previous studies have shown that TGF-β1 promotes the remodeling and deposition of ECM by activating downstream target genes such as MMPs and TIMPs. The TGF-β1-mediated signaling pathway depends on the phosphorylation of Smad2/3, which has been reported as a potential target for antifibrotic therapy [12–14, 27]. To understand the molecular mechanisms responsible for the inhibition of HSC activation, the protein activity of Smad2/3 (intracellular mediators of TGF-β1 signal transduction) was assessed. Western blot analysis showed significant increases in the phosphorylation of Smad2/3 stimulated by TGF-β1, and casticin treatment decreased the enhancement of phosphorylation by TGF-β1 (Figure 5B). We then confirmed that casticin inhibited the fibrotic effects of TGF-β1 on ECM deposition in LX2 cells by evaluating the mRNA levels of TGF-β, collagen α1(I), MMP-2, MMP-9, TIMP-1 and TIMP-2. Real-time PCR revealed that TGF-β1 significantly increased TGF-β, collagen α1(I), MMP-2, MMP-9, TIMP-1 and TIMP-2, but casticin reversed these trends (Figure 5C–5H). These results demonstrate that casticin inhibited TGF-β1-induced HSC activation and ECM remodeling.

### Casticin inhibits HSC activation and collagen matrix expression by blocking TGF-β/Smad signaling *in vivo*

**Figure 6:**
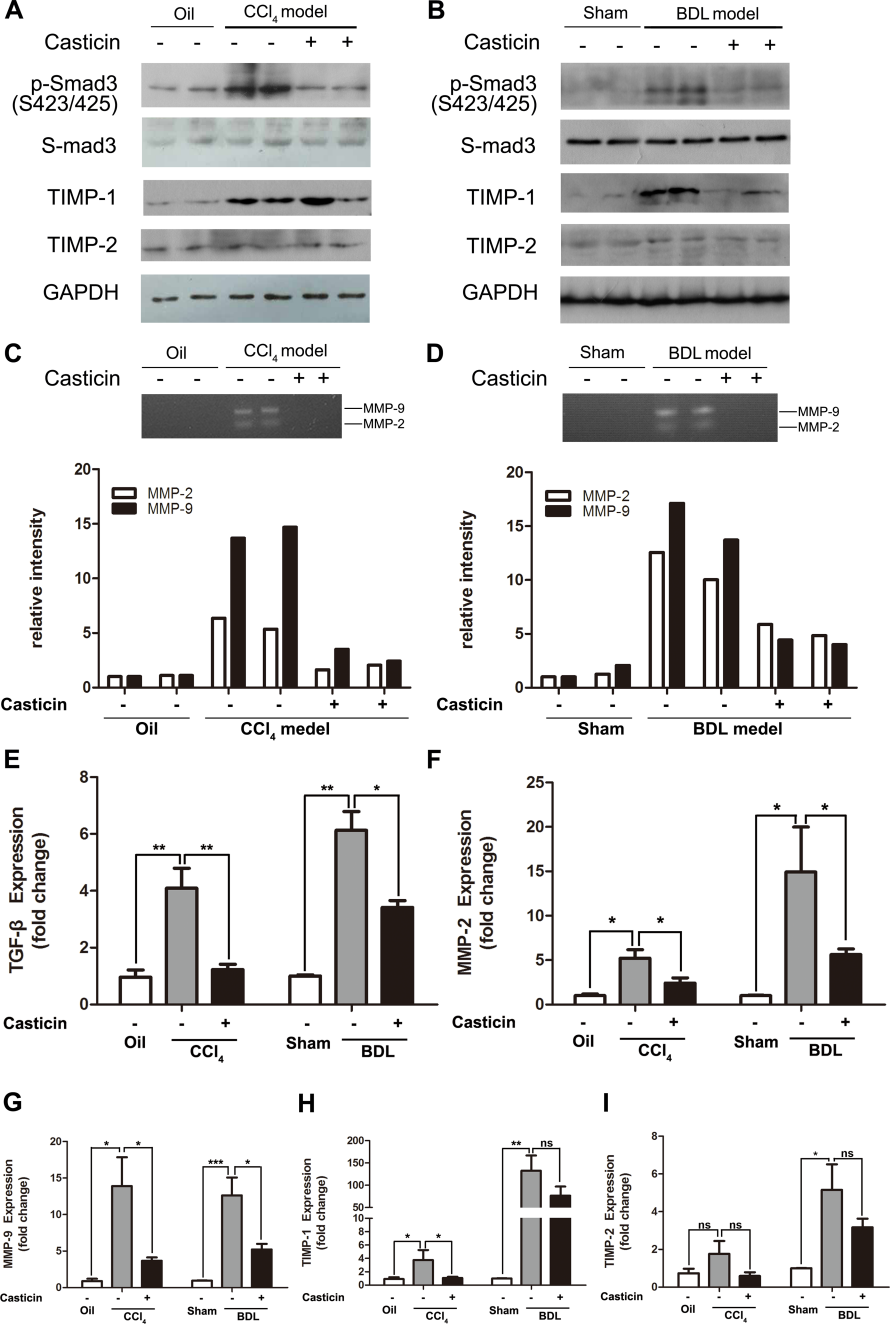
Effects and probable mechanisms of casticin on HSC activation *in vivo* (**A**–**B**) Following treatment as previously described, the mice were executed, and their liver tissue were lysed and subjected to Western blot analysis to assess p-Smad3(S423/425), TIMP-1 and TIMP-2. Casticin administration decreased the phosphorylation of Smad3 and the level of TIMP-1. However, the protein expression of TIMP-2 did not show any difference between groups. (**C**–**D**) Zymography of liver lysates showing MMP-2 and MMP-9 gelatinase activity (upper panel) with densitometric quantification (lower panel). (**E**) Hepatic mRNA levels for TGF-β1 were measured by real-time RT-PCR. (**F**–**I**). Hepatic mRNA levels for MMP-2, MMP-9, TIMP-1 and TIMP-2 were measured by real-time RT-PCR.

Finally, we identified potential antifibrotic mechanisms of casticin *in vivo*. Given that casticin can efficiently inhibit TGF-β1-induced HSC activation, we investigated the mechanism by which casticin attenuates CCl_4_-or BDL-induced liver fibrosis *in vivo*. As shown in Figure 6A and 6B, compared with control mice, CCl_4_-or BDL-induced liver fibrosis was accompanied by a marked activation of p-Smad3 (S423/425) and upregulation of TGF-β1 mRNA expression (Figure 6E), while casticin administration significantly decreased the phosphorylation of Smad3 and the level of TGF-β1 mRNA.

The inhibitory function of casticin on the pro-fibrotic effects of TGF-β1 on ECM accumulation was further examined. Previous studies have demonstrated the MMPs and their endogenous TIMP inhibitors played a key role in ECM remodeling. Thus we first detected the serum MMP-2 and pro MMP-9 by Elisa, and the data illustrated that serum MMP-2 and pro MMP-9 was increased in the mice induced by CCl_4_ or BDL, but they were evidently decreased following casticin treatment (Supplementary Figure 2A–2B). Then to monitor the functional MMP-activity, gelatinase zymography have been performed. Compared to the mice induced by CCl_4_ or BDL, casticin administration showed a prominently decreased gelatinase activity (Figure 6C–6D). Real-time PCR test indicated the same trend in the mRNA expression of MMP-2, MMP-9, TIMP-1 and TIMP-2 (Figure 6F–6I). These results confirmed that casticin ameliorated experimental hepatic fibrosis by inhibiting HSC activation and collagen matrix accumulation through TGF-β/Smad signaling.

## DISCUSSION

Hepatic fibrosis is a general consequence of chronic liver disease, and is characterized by a multicellular response with the activation of HSCs as a critical component. Therefore, inhibition of the accumulation of activated HSCs by modulating either their activation and/or proliferation, or by promoting HSC apoptosis, is the main target in patients with hepatic fibrosis [5]. Recently, much interest in herbal medicine has been focused on the hepatoprotective or antifibrotic effects of compounds such as phyllanthus, silymarin, glycyrrhizin and sho-saiko-to [30]. In this study, we first demonstrate that casticin has an inhibitory effect on liver fibrosis *in vitro* and *in vivo* as evidenced by the alleviation of fibrosis-related injury accompanied by reductions in collagen deposition and the number of α-SMA-positive cells, as well as diminished expression of profibrogenic markers in mouse fibrotic liver induced by CCl_4_ or BDL.

Casticin is one of the main components derived from the dried fruits of *Vitex rotundifolia L*. and has been used in prescriptions of traditional medicine for the treatment of colds, headache, migraine, sore eyes and other ailments. In 2004, Kobayakaw and colleagues confirmed that casticin disrupted mitotic spindles and showed that it had antimitotic effects on the human epidermoid carcinoma KB cell line without influencing the growth of normal cells [19]. They suggested that G2-M arrest by casticin may inhibit microtubule dynamics. On this basis, casticin has been shown to inhibit the proliferation of breast cancer, lung cancer, colon cancer, human myelogenous leukemia cells and hepatocellular carcinoma cells *in vitro* [20–24, 31–33]. These findings suggest a similar anti-apoptotic inhibitory effect of casticin on rapid-proliferating cell types. In 2009, Choudhary and colleagues first reported the anti-inflammatory activities of casticin *in vitro*, and suggested their potential as non-steroidal anti-inflammatory agents [34]. Recently, several studies have demonstrated that the anti-inflammatory effect of casticin is due to inhibition of pro-inflammatory cytokines and inflammatory mediators including NO and PGE2, blocking in turn the activation of NF-κB, Akt, and MAPK signaling [26]. However, almost no information is available regarding the anti-fibrotic effects of casticin on liver fibrosis. Our *in vivo* study using two mouse models of liver fibrosis induced by carbon tetrachloride and bile duct ligation indicated that casticin exerted hepatoprotective and anti-fibrotic effects. *In vitro*, we also showed that casticin could inhibit the proliferation and induce apoptosis in the LX2 human hepatic stellate cell line. Our results were consistent with the previous studies that showed the casticin have key roles in regulating cell proliferation and apoptosis.

The molecular mechanism of cell activation in HSCs has recently been extensively investigated [1–6]. In our study, we characterized inhibition of TGF-β1 signaling most likely plays an essential role in the anti-fibrotic effect of casticin. Since TGF-β1 is one of the key regulators of HSC activation [1], it is also possible that casticin inhibits autocrine/paracrine signaling in activated HSCs, thereby facilitating resolution of CCl_4_- or BDL-induced hepatic fibrosis *in vivo*. In addition, it has been postulated that the constitutive activation of TGF-β1 signaling is one of the main factors acting as a cell-survival signal in activated HSCs [1]. Indeed, most chemicals which suppress the activation of HSCs have been shown to inhibit TGF-β1 signaling [35–36]. Our studies concluded the same results. Furthermore, our experiments also indicated the casticin had the pro-apoptotic effect on HSCs. It has been reported that casticin could induce apoptosis through NF-κB, ASK1-JNK-Bim and mitotic arrest in many types of tumour cells [19–24], however, it was unclear the apoptosis could be induced by TGF-β/Smad signaling pathway especially in the activated HSCs . Although our study indicated these results, we could not make sure to draw this conclusion. Further experiments need to be carried on.

In conclusion, casticin suppresses the activation of HSCs and induces apoptosis in activated HSCs both *in vitro* and *in vivo*. Although a complete understanding of the molecular mechanisms will require further investigation, it is likely that casticin inhibits TGF-β1 signaling pathways, leading to a reduction in downstream phosphorylation of Smad2/3 that inhibits HSC activation. Future approaches examining clinical applications of casticin are promising for the establishment of a new treatment for hepatic fibrosis in a variety of chronic liver diseases.

## MATERIALS AND METHODS

### Materials

Sirius red was obtained from Sigma-Aldrich (St. Louis, MO, USA). CCl_4_ was purchased from Aladdin Industrial Corporation (Shanghai, China). Casticin was purchased from Chengdu Biopurify Phytochemicals Ltd. (Chengdu, China). Casticin has a molecular weight of 374.3 kDa, appears as yellow crystals and had a purity of 98.0%. Antibodies against glyceraldehyde-3-phosphate dehydrogenase (GAPDH) and HRP-conjugated anti-mouse and anti-rabbit IgG were obtained from Sigma-Aldrich. Antibodies against vimentin, phospho-Smad2 (S465/467), phospho-Smad3 (S423/425) and poly (adenosine diphosphate-ribose) polymerase (PARP), TIMP-1 and TIMP-2 were purchased from Cell Signaling Technology (Danvers, MA, USA), and antibody against α-SMA was purchased from Abclonal Technology (Wuhan, China).

### Animal models of liver fibrosis

### CCl_4_ intraperitoneal injection

Male mice 6–8 weeks of age weighing 20–30 g were purchased from Southern Medical University Experimental Animal Center (Guangzhou, China) and kept in a temperature-controlled room with an alternating 12 h dark and light cycle. All procedures involving animals in this study were approved by the animal care committee of Southern Medical University in accordance with institutional guidelines for animal experiments. A total of 32 mice were divided randomly into four groups of 8 animals each: control, casticin, CCl_4_, and CCl_4_ + casticin. To induce liver fibrosis, CCl_4_ dissolved in olive oil (20%) was injected intraperitoneally into mice (1.0 ml/kg body weight) in the CCl_4_ and CCl_4_ + casticin groups twice a week for six weeks. Mice in the control group and casticin group were injected with an equivalent volume of olive oil. Casticin was dissolved in 0.25% Tween-80. After treatment with CCl_4_ or olive oil for six weeks, mice in the casticin group and CCl_4_ + casticin group received casticin (20 mg/kg) by gastric gavage daily for two weeks, and the other two groups were given the equivalent volume of 0.25% Tween-80. After the eight week intervention period, mice were euthanatized under 3% pentobarbital sodium anesthesia (40 mg/kg ip), and the livers and blood from all animals were collected. Serum was obtained by centrifugation (1600 g, 15 min) and stored at −20°C for further examination.

### Bile duct ligation surgery

Male mice 6–8 weeks of age weighing 20–30 g underwent either sham operation or bile duct ligation (BDL) [37]. A total of 32 mice were divided randomly into four groups of 8 animals each: control, casticin, BDL, and BDL + casticin. In sham operation, the bile duct was exposed, but not ligated. After BDL for 4 days, mice were administered intragastrically with casticin (*n* = 8) at 20 mg/kg/day or 0.25% Tween-80 (*n* = 8) for 2 weeks. Liver samples from these mice were collected within 24 h after the last administration of casticin. The samples were snap-frozen in liquid nitrogen and stored at −80°C until used.

### Serum aminotransferases, albumin and total bilirubin

Serum levels of alanine aminotransferase (ALT), aspartate aminotransferase (AST), albumin and total bilirubin were determined by Catalyst Dx^™^ Chemistry Analyzer (Yeeran Technology Limited, Beijing, China).

### Serum MMP-2 and pro MMP-9 determination

Mouse MMP-2 and pro MMP-9 Elisa Kit are the enzyme-linked immunosorbent assay for measuring mouse MMP-2 and pro MMP-9 in serum. Mouse MMP-2 Elisa Kit and Mouse pro MMP-9 Elisa Kit were purchased from Thermo Scientific (Frederick, USA).

### Liver histology and morphometric collagen determination

The livers were fixed in 4% paraformaldehyde and embedded in paraffin, sectioned at 4 μm and stained with hematoxylin-eosin (H&E) and sirius red. The extent of fibrosis was evaluated on blinded slides by a member of our Department of Pathology. Fibrosis was determined histologically by measuring the intensity of fibrosis in five (magnification 100×) digital images captured from slides of each mouse stained with sirius red. The total fibrosis density score was determined by dividing the image intensity by the image area as described in reference [38].

### Determination of hepatic hydroxyproline content

The hepatic hydroxyproline content as an indirect measure of tissue collagen content was expressed as μg/gram of liver wet weight. It was measured by using a hydroxyproline detection kit (Jiancheng Institute of Biotechnology, Nanjing, China) according to the manufacturer's instructions.

### Gelatin zymography

MMP-2, 9 activity were assessed by gelatin zymography as described before [39–40]. For gelatin zymography 1 mg/mL gelatin was copolymerized in a 7.5% PAGE gel and 20 μg protein of pooled liver lysates of each group was loaded.

### Immunohistochemistry

After deparaffinization and dehydration, microwave antigen retrieval was performed, followed by peroxidase quenching. Subsequently, the sections were blocked with goat serum for 1 h at room temperature and incubated overnight at 4°C with 1:100 dilutions of primary antibodies to α-SMA. Negative control sections were treated identically, except for omission of the primary antibodies. After washing in PBS, the sections were incubated with HRP-conjugated secondary antibodies for 1 h at room temperature, and immunoreactivity was visualized with 3,3′-Diaminobenzidine (DAB) staining. Finally, slides were counterstained with Harris hematoxylin. For quantitation of immunoreactivity, 15 consecutive nonoverlapping fields at 200× magnification were scored using a graticule eyepiece in a blinded fashion.

### Cell culture

LX 2 cells, an immortalized human HSC cell line, were purchased from KeyGEN BioTECH (Jiangsu, China). Cells were cultured in RPMI-1640 medium supplemented with 10% fetal bovine serum and incubated at 37°C in a humidified air atmosphere containing 5% CO_2_. Morphological changes after indicated treatments were observed by regular phase contrast microscope.

To evaluate the effect of casticin on LX-2 cells using real-time PCR and western blot analysis, cells were serum-starved overnight, then treated with 10 ng/μl TGF-β1 for 30–60 min in the presence or absence of casticin for the indicated time periods (0.5–12 h). The casticin was dissolved with dimethylsulfoxide (DMSO) , then diluted to determined concentration with culture medium.

### Cell proliferation assay

LX-2 cells or L02 cells were plated at a density of 5 × 10^3^ cells per well in a 96-well plate and treated with casticin (0–40 μM) for 48 h in growth medium containing serum. Cell proliferation was determined using a CCK-8 assay kit (Dojindo, Tokyo, Japan) according to the manufacturer's instructions.

### Apoptosis analysis

To quantitatively assess the rate of apoptosis, AV-PI staining was conducted. We used Annexin V/propidium iodide double-labelled flow cytometry kit (KeyGen, Nanjing, China) as the manufacturer described. Briefly, 2 × 10^5^ LX2 cells or L02 cells were treated with casticin (0–40 μM) for 12 h, respectively. After harvested and washed twice with PBS, the cells were resuspended in 500 μl binding buffer. Then, 5 μl of annexin V and 5 μl of PI were added. The mixture was incubated at room temperature for 5 min in the dark. Then cells were analysed by flow cytometry with a Becton Dickinson FACS-420 flow cytometer (Franklin Lakes, NJ, USA).

### Immunofluorescence

LX-2 cells were grown on chamber slides and treated with casticin and TGF-β1 as previously described. The cells were fixed in 4% paraformaldehyde/PBS (10 min) and then incubated in 1% BSA/10% normal goat serum in 0.1% PBS-Tween for 1 h to permeabilize the cells and block non-specific protein interactions. The cells were then incubated with antibody against α-SMA at 10 μg/ml overnight at 4°C. The secondary antibody (red) was Alexa Fluor^R^ 594-labeled goat anti-mouse IgG used at a 1/500 dilution for 1 h. Diamidino-phenyl-indole (DAPI) was used to stain the cell nuclei (blue) at a concentration of 1.43 μM. After washing with PBS four times for 15 min, cells were mounted and analyzed by fluorescence microscopy (magnification 200×).

### Gene expression analysis by quantitative real-time PCR

Total RNA was isolated from liver tissue in mice and from LX-2 cells using Trizol reagent (Invitrogen, Carlsbad, CA, USA). RNA (1 μg) was reverse-transcribed using a reverse transcription-polymerase chain reaction (RT-PCR) kit (Promega, Madison WI, USA) to obtain cDNA. Real-time PCR was carried out using the 7500 real-time PCR system (Applied Biosystems, Foster City, CA, USA) using a SYBR Green PCR Kit (TaKaRa, Shiga Prefecture, Japan). The Cp value of α1(I) collagen, TGF-β1, MMP-2, MMP-9, TIMP-1 and TIMP-2 was normalized based on that of GAPDH using 7500 System SDS Software Version 1.2 (Applied Biosystems). A melting curve analysis was done after amplification to verify the accuracy of the amplicon. The primer pairs used were listed in Table 1.

**Table 1:** Primer sequences and accession numbers for primers used for RT-PCR

Gene	Accession No.	Forward Primer (5′–3′)	Reverse Primer (5′–3′)
*co*l1α1	NM_007742.3	GCTCCTCTTAGGGGCCACT	CCACGTCTCACCATTGGGG
*TGF*-β1	NM_011577.4	CTCCCGTGGCTTCTAGTGC	GCCTTAGTTTGGACAGGATCTG
*TIMP1*	NC_000086.7	GCATCTCTGGCATCTGGCATC	TGACGTCACTGGAGTTGTACGG
*TIMP2*	NM_011594.1	TCAGAGCCAAAGCAGTGAGC	GCCGTGTAGATAAACTCGATGTC
*MMP-2*	NC_000074.6	TTCCCCCGCAAGCCCAAGTG	GAGAAAAGCGCAGCGGAGTGACG
*MMP-*9	NC_000068.7	CTGGACAGCCAGACACTAAAG	CTCGCGGCAAGTCTTCAGAG

### Western blot analysis

Cells were lysed in 2×SDS sample buffer (adding protease inhibitors and phosphatase inhibitor) on ice. Equal amounts of whole protein extract were resolved by SDS-PAGE, transferred to a nitrocellulose membrane and probed overnight at 4°C with primary antibodies. Membranes were washed with TBS/0.05% Tween-20 and incubated with HRP-conjugated secondary antibodies at room temperature for 1 h. Proteins were detected by enhanced chemiluminescence substrates (PerkinElmer, Massachusetts, USA).

### Statistical analysis

All data were expressed as mean±SEM Student's *t* test or two-way analysis of variance was used to analyze differences between groups. A value of *P* < 0.05 was considered statistically significant.

## SUPPLEMENTARY MATERIALS FIGURES



## Abbreviations

CCl_4_: carbon tetrachloride
α-SMA: α-smooth muscle actin
PARP: poly (adenosine diphosphate-ribose) polymerase
HSC: hepatic stellate cell
MMP: matrix metalloproteinase
TIMP: tissue inhibitor of metalloproteinases
TGF-β1: transforming growth factor beta1
ECM: extracellular matrix
ALT: alanine transaminase
AST: aspartate aminotransferase
DAB: 3,3′-Diaminobenzidine
DAPI: diamidino-phenyl-indole
RT-PCR: real time-polymerase chain reaction
Col1α1: collagen type1 alpha 1.

## References

[1] Hernandez-Gea V, Friedman SL (2011). Pathogenesis of liver fibrosis. Annu Rev Pathol.

[2] Bataller R, Brenner DA (2005). Liver fibrosis. J Clin Invest.

[3] Kisseleva T, Brenner DA (2008). Fibrogenesis of parenchymal organs. Proc Am Thorac Soc.

[4] Tsochatzis EA, Bosch J, Burroughs AK (2014). Liver cirrhosis. Lancet.

[5] Schuppan D, Kim YO (2013). Evolving therapies for liver fibrosis. J Clin Invest.

[6] Zhang DY, Friedman SL (2012). Fibrosis-dependent mechanisms of hepatocarcinogenesis. Hepatology.

[7] Trautwein C, Friedman SL, Schuppan D, Pinzani M (2015). Hepatic fibrosis: Concept to treatment. J Hepatol.

[8] Friedman SL (2008). Mechanisms of hepatic fibrogenesis. Gastroenterology.

[9] Lee YA, Wallace MC, Friedman SL (2015). Pathobiology of liver fibrosis: a translational success story. Gut.

[10] Bataller R, Brenner DA (2001). Hepatic stellate cells as a target for the treatment of liver fibrosis. Semin Liver Dis.

[11] Border WA, Noble NA (1994). Transforming growth factor beta in tissue fibrosis. N Engl J Med.

[12] Gressner AM, Weiskirchen R, Breitkopf K, Dooley S (2002). Roles of TGF-beta in hepatic fibrosis. Front Biosci.

[13] Breitkopf K, Godoy P, Ciuclan L, Singer MV, Dooley S (2006). TGF-β/Smad Signaling in the Injured Liver. Zeitschrift für Gastroenterologie.

[14] Fabregat I, Moreno-Caceres J, Sanchez A, Dooley S, Dewidar B, Giannelli G, Ten DP (2016). TGF-beta Signaling and Liver Disease. FEBS J.

[15] Cales P (1998). Apoptosis and liver fibrosis: antifibrotic strategies. Biomed Pharmacother.

[16] Li J, Li X, Xu W, Wang S, Hu Z, Zhang Q, Deng X, Wang J, Zhang J, Guo C (2015). Antifibrotic effects of luteolin on hepatic stellate cells and liver fibrosis by targeting AKT/mTOR/p70S6K and TGFbeta/Smad signalling pathways. Liver Int.

[17] Chan CC, Cheng LY, Lin CL, Huang YH, Lin HC, Lee FY (2011). The protective role of natural phytoalexin resveratrol on inflammation, fibrosis and regeneration in cholestatic liver injury. Mol Nutr Food Res.

[18] Wang X, Ikejima K, Kon K, Arai K, Aoyama T, Okumura K, Abe W, Sato N, Watanabe S (2011). Ursolic acid ameliorates hepatic fibrosis in the rat by specific induction of apoptosis in hepatic stellate cells. J Hepatol.

[19] Kobayakawa J, Satonishimori F, Moriyasu M, Matsukawa Y (2004). G2-M arrest and antimitotic activity mediated by casticin, a flavonoid isolated from Viticis Fructus (Vitex rotundifolia Linne fil.). Cancer Lett.

[20] Shen J, Du H, Yang M, Wang Y, Jin J (2009). Casticin induces leukemic cell death through apoptosis and mitotic catastrophe. Ann Hematol.

[21] Yang J (2011). Casticin-induced apoptosis involves death receptor 5 upregulation in hepatocellular carcinoma cells. World J Gastroentero.

[22] Chen D, Cao J, Tian L, Liu F, Sheng X (2011). Induction of apoptosis by casticin in cervical cancer cells through reactive oxygen species-mediated mitochondrial signaling pathways. Oncol Rep.

[23] Rasul A, Zhao B, Liu J, Liu B, Sun J, Li J, Li X (2014). Molecular Mechanisms of Casticin Action: an Update on its Antitumor Functions. Asian Pac J Cancer P.

[24] Haïdara K, Zamir L, Shi Q, Batist G (2006). The flavonoid Casticin has multiple mechanisms of tumor cytotoxicity action. Cancer Lett.

[25] Li Y, Guo Y, Yang Q, Weng X, Yang L, Wang Y, Chen Y, Zhang D, Li Q, Liu X, Kan X, Chen X, Zhu X (2015). Flavonoids casticin and chrysosplenol D from Artemisia annua L. inhibit inflammation in vitro and in vivo. Toxicol Appl Pharm.

[26] Lee H, Jung K, Lee H, Park S, Choi W, Bae H (2015). Casticin, an active compound isolated from Vitex Fructus, ameliorates the cigarette smoke-induced acute lung inflammatory response in a murine model. Int Immunopharmacol.

[27] Wynn TA (2008). Cellular and molecular mechanisms of fibrosis. J Pathol.

[28] Oliver FJ, de la Rubia G, Rolli V, Ruiz-Ruiz MC, de Murcia G, Murcia JM (1998). Importance of poly(ADP-ribose) polymerase and its cleavage in apoptosis. Lesson from an uncleavable mutant. J Biol Chem.

[29] Satoh MS, Lindahl T (1992). Role of poly(ADP-ribose) formation in DNA repair. Nature.

[30] Levy C, Seeff LD, Lindor KD (2004). Use of herbal supplements for chronic liver disease. Clin Gastroenterol Hepatol.

[31] He M, Cao XC, He GC, Sheng XF, Ai XH, Wu YH (2014). Casticin inhibits epithelial-mesenchymal transition of liver cancer stem cells of the SMMC-7721 cell line through downregulating Twist. Oncol Lett.

[32] He G, Cao X, He M, Sheng X, Wu Y, Ai X (2014). Casticin inhibits self-renewal of liver cancer stem cells from the MHCC97 cell line. Oncol Lett.

[33] Liu F, Cao X, Liu Z, Guo H, Ren K, Quan M, Zhou Y, Xiang H, Cao J (2013). Casticin suppresses self-renewal and invasion of lung cancer stem-like cells from A549 cells through down-regulation of pAkt. Acta Bioch Bioph Sin.

[34] Choudhary MI, Jalil S, Nawaz SA, Khan KM, Tareen RB (2009). Anti inflammatory and lipoxygenase inhibitory compounds from Vitex agnus-castus. Phytother Res.

[35] Xu T, Ni MM, Xing-Li, Li XF, Meng XM, Huang C, Li J (2016). NLRC5 regulates TGF-β1-induced proliferation and activation of hepatic stellate cells during hepatic fibrosis. Int J Biochem Cell Biol.

[36] Ganai AA, Husain M (2016). Genistein attenuates D-GalN induced liver fibrosis/chronic liver damage in rats by blocking the TGF-β/Smad signaling pathways. Chem Biol Interact.

[37] Tag CG, Sauer-Lehnen S, Weiskirchen S, Borkham-Kamphorst E, Tolba RH, Tacke F, Weiskirchen R (2015). Bile duct ligation in mice: induction of inflammatory liver injury and fibrosis by obstructive cholestasis. J Vis Exp.

[38] Potter JJ, Rennie-Tankesley L, Mezey E (2003). Influence of leptin in the development of hepatic fibrosis produced in mice by Schistosoma mansoni infection and by chronic carbon tetrachloride administration. J Hepatol.

[39] Hu X, Beeton C (2010). Detection of Functional Matrix Metalloproteinases by Zymography. JoVE.

[40] Snoek-van Beurden PA, Von den Hoff JW (2005). Zymographic techniques for the analysis of matrix metalloproteinases and their inhibitors. Biotechniques.

