# Supplementary material for: Casticin attenuates liver fibrosis and hepatic stellate cell activation by blocking TGF-β/Smad signaling pathway
Source: Oncotarget. 2017 Apr 27;8(34):56267–80. doi: 10.18632/oncotarget.17453 (PMC5593560; doi:10.18632/oncotarget.17453)
Supplement: Supplementary file 1 [file oncotarget-08-56267-s001.pdf]

Casticin attenuates liver fibrosis and hepatic stellate cell activation by blocking TGF-β/Smad signaling pathway

Supplementary Materials

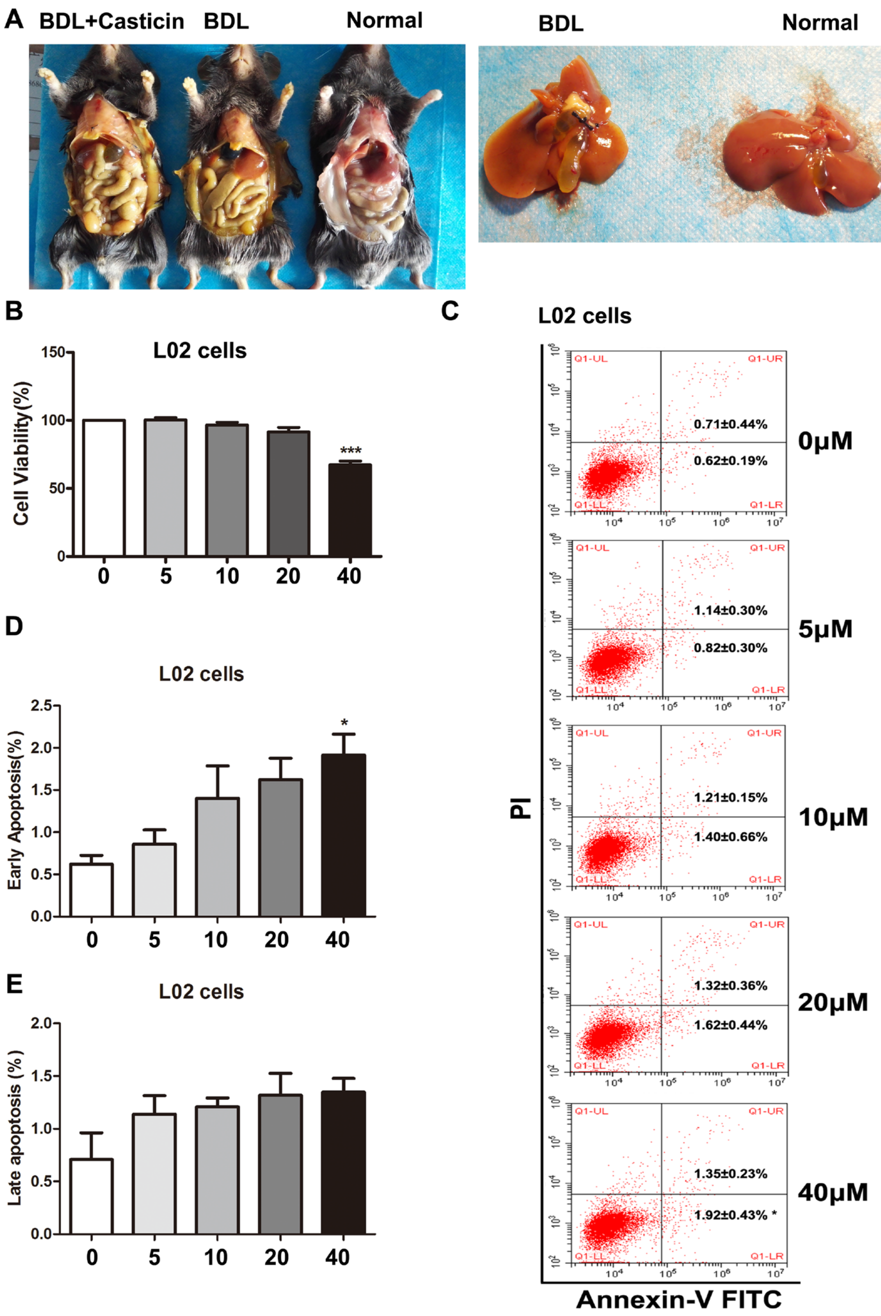

Supplementary Figure 1: (**A**) Representative appearance of livers 2 weeks after sham operation and BDL. (**B–E**) Effect of casticin on cell proliferation and apoptosis of L02 cells.

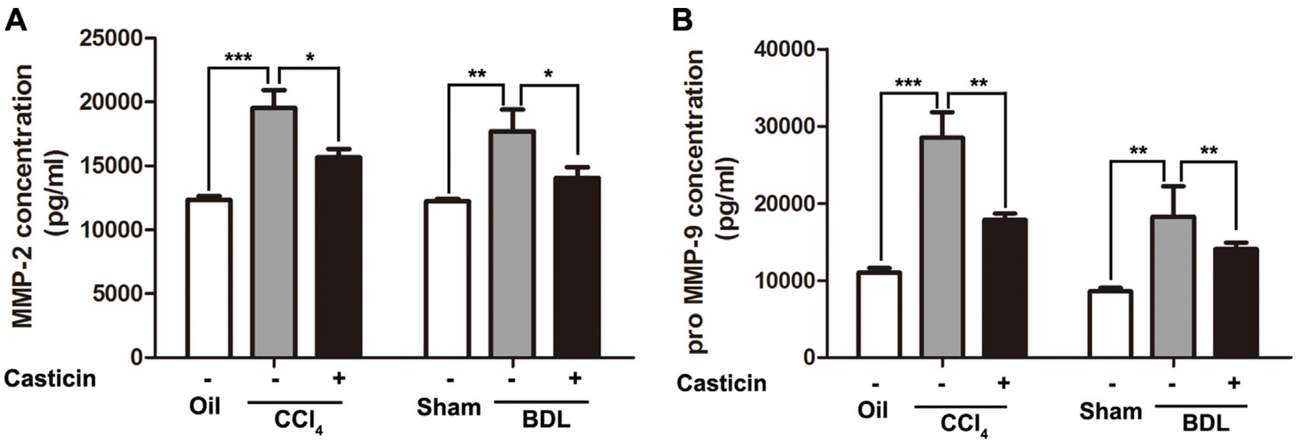

Supplementary Figure 2: (**A**–**B**) Serum MMP-2 and pro MMP-9 determination were detected by Elisa.
